# Comparison of SOX and CAPOX in patients with advanced gastric cancer after laparoscopic D2 gastrectomy: A randomized controlled trial

**DOI:** 10.1002/cam4.7326

**Published:** 2024-06-03

**Authors:** Xin Liu, Yongjia Yan, Li Lu, Yang Liu, Jun Ma, Xi Wang, Daohan Wang, Bang Liu, Zhuo Liu, Xueying Zhou, He Cui, Zhicheng Zhao, Chuan Li, Jian Liu, Weidong Li, Qing‐Xing Huang, Qun Zhao, Tong Liu, Weihua Fu

**Affiliations:** ^1^ Department of General Surgery Tianjin Medical University General Hospital Tianjin China; ^2^ Department of General Surgery the Fourth Affiliated Hospital, Hebei Medical University Shijiazhuang China; ^3^ Department of Digestive Endoscopic and Minimally Invasive Surgery Shanxi Cancer Hospital Taiyuan China

**Keywords:** chemotherapy, gastric cancer, surgery, survival

## Abstract

Background: Optimal adjuvant chemotherapy after laparoscopic surgery in gastric cancer (GC) patients is still undefined. We aimed to evaluate the efficacy of S‐1 plus oxaliplatin (SOX) and capecitabine plus oxaliplatin (CAPOX) in patients with GC after laparoscopic gastrectomy. Methods: A non‐inferiority randomized controlled clinical trial was performed in China. Patients with advanced GC who underwent laparoscopic D2 gastrectomy were randomly assigned to receive SOX and CAPOX regimens. Results: In total, 191 patients were screened between May 2018 and June 2019, and 140 (73.3%) were included in the modified intent‐to‐treat analysis (mITT), of whom 69 and 71 were assigned to the SOX and CAPOX groups, respectively. The SOX group had similar 3‐year overall survival (OS) and disease‐free survival to the CAPOX group. Subgroup analysis revealed significantly better OS in the SOX group for male patients ([HR] = 0.395; 95% [CI], 0.153–1.019; *p* = 0.045), age >60 (HR = 0.219; 95% [CI], 0.064–0.753; *p* = 0.016), tumors in the gastric antrum (HR = 0.273; 95% [CI], 0.076–0.981; *p* = 0.047), and moderately differentiated tumors (HR = 0.338; 95% [CI], 0.110–1.041; *p* = 0.041). There were no significant differences observed in terms of adverse events and recurrence patterns between the two groups. Conclusion: Adjuvant SOX was non‐inferior to CAPOX treatments for patients with GC who underwent curative laparoscopic D2 gastrectomy. For male patients, aged >60 years, tumors in the gastric antrum, and moderately differentiated tumors, adjuvant SOX may achieve an improvement compared with CAPOX.

## Graphical abstract

A prospective randomized controlled trial was performed to evaluate the efficacy of S‐1 plus oxaliplatin (SOX) and capecitabine plus oxaliplatin (CAPOX) regimens for patients with advanced gastric cancer (GC) who underwent laparoscopic D2 radical gastrectomy. In the subgroup analysis, the group of patients with male sex, age >60 years, tumors in the gastric antrum, and moderately differentiated tumors showed significantly better overall survival for the SOX regimen.

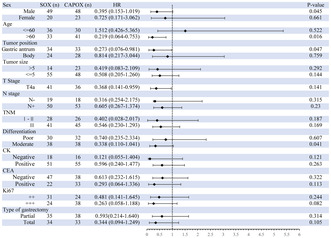

## 1 INTRODUCTION

Gastric cancer (GC) ranks among the prevalent malignancies globally, with a particularly high incidence in Asian nations. According to the World Health Organization, there were approximately 1.09 million new cases and over 760,000 deaths from GC in 2020.^1^ Radical gastrectomy with D2 lymphadenectomy has been considered the optimal treatment for resectable GC.^2^, ^3^, ^4^ However, approximately 50% of GC patients experience recurrence after complete resection.^5^ Therefore, surgical treatment should be combined with other treatment modalities to consolidate the therapeutic effects of surgery.

A synthesis of randomized controlled trials indicated that fluorouracil‐based adjuvant chemotherapy regimens could increase 5‐year overall survival (OS) from 49.6% to 55.3% compared with surgical excision alone.^6^ In ACTS‐GC, the efficacy of S‐1 was evaluated as an adjuvant treatment after surgery for patients with Stage II/III GC.^5^ However, the efficacy of monotherapy was not obvious in patients with Stage IIIA disease, and especially not in patients with Stage IIIB disease. Thus, combination chemotherapy has gradually become the first‐line postoperative chemotherapy for patients with GC. In the ARTIST 2 trial, the 3‐year DFS rate was remarkably improved by SOX regimens compared to S‐1 chemotherapy.^7^ In the CLASSIC trial, the 3‐year DFS rate was 74% in group receiving the regimen of capecitabine and oxaliplatin (CAPOX), which was also significantly improved compared to surgery alone (59%).^8^ Hence, SOX and CAPOX have been considered as the preferred chemotherapy regimens in Eastern and Western countries. In a prospective study (RESOLVE),^9^ SOX was proven to be non‐inferior to CAPOX in patients with locally advanced GC. Hence, the integration of surgery with chemotherapy is now acknowledged as the primary treatment approach for resectable gastric cancer.

The application of laparoscopy in gastrointestinal surgery is becoming increasingly extensive. In the CLASS‐01^10^ and KLASS‐02^11^ trials, laparoscopic D2 radical gastrectomy was proven safer than open surgery. However, laparoscopy also has limitations. The establishment of pneumoperitoneum may increase the risk of tumor cell dissemination and implantation in the abdominal cavity.^12^, ^13^, ^14^ Previous comparisons of postoperative chemotherapy regimens for GC did not focus on the distinction between surgical methods. It was necessary to find an appropriate chemotherapy regimen for patients who underwent laparoscopic gastrectomy.

In this study, we conducted a prospective randomized controlled trial to assess the effectiveness of SOX and CAPOX regimens in patients with advanced gastric cancer who underwent laparoscopic D2 radical gastrectomy.

## 2 METHODS

### 2.1 Study design and patients

This multicenter study was prospective, randomized, and comprised of two arms. It was conducted at three different centers in China. Our objective was to evaluate the efficacy of SOX and CAPOX in patients with advanced GC who underwent D2 gastrectomy. The study was a non‐inferiority trial. The primary endpoint was a 3‐year OS. The secondary endpoint was a 3‐year DFS and chemotherapy‐related adverse events. The reporting of this study adheres to the guidelines outlined in the Consolidated Standards of Reporting Trials (CONSORT).^15^ Following the screening process based on inclusion and exclusion criteria, according to a 1:1 randomization, participants were assigned to either the CAPOX group or the SOX group after the surgery. The randomization scheme was generated by a statistician using a randomization algorithm, and the researchers were responsible for enrolling eligible participants according to the scheme. When a participant met the inclusion criteria, the corresponding treatment regimen was implemented by the physician. Data analysis was performed by blinded evaluators.

Between May 2018 and June 2019, 144 patients who had undergone laparoscopic gastrectomy with R0 resection and D2 lymph node dissection were randomly assigned to the SOX and CAPOX groups. The inclusion criteria were Stage IB–III, histologically confirmed diagnosis of adenocarcinoma, age 20–80 years, no history of chemoradiotherapy, no bulky lymph node metastasis, and no emergency indication for surgery. Every participant provided their consent to take part in the study and formally signed informed consent forms.

The study protocols and consent forms were approved by the institutional review boards of all the participating institutions. The trial was carried out in compliance with the principles stated in the Declaration of Helsinki (2002) and in accordance with the regulations governing clinical trial research in China.

### 2.2 Treatment and assessment

Surgical procedures are shown in Doc. S1. After surgery, patients underwent routine treatment and nursing sessions. Chemotherapy began within 2–4 weeks after surgery according to the general condition of the patients. Based on the results of previous studies,^8^, ^16^ chemotherapy regimens were formulated as follows. The patients received SOX or CAPOX regimens for eight cycles with each treatment cycle lasting 21 days. The SOX regimen consisted of oxaliplatin (130 mg/m^2^ administered intravenously on Day 1 of each cycle) plus S‐1 (60 mg administered orally twice daily on Days 1–14 of each cycle). The CAPOX regimen consisted of oxaliplatin (130 mg/m^2^ administered intravenously on Day 1 of each cycle) plus capecitabine (1000 mg/m^2^ administered orally twice daily on Days 1–14 of each cycle). When a patient experienced Grade 3–4 adverse reactions for the first time during chemotherapy, we adopted corresponding symptomatic treatment, such as giving recombinant human granulocyte colony‐stimulating factor to patients with myelosuppression, and suspending chemotherapy. If the patient experienced the adverse reactions for the second time, the chemotherapy regimen should be reduced or replaced with an alternative chemotherapy regimen. The alternative treatment regimen was the FOLFOX regimen, comprising oxaliplatin (85 mg/m^2^ on Day 1), leucovorin (400 mg/m^2^ on Day 1), and 5‐fluorouracil (400 mg/m^2^ on Day 1, 2400 mg/m^2^ on Days 2–3) administered every 3 weeks. Upon completion of chemotherapy, patients underwent follow‐up assessments every 3 months during the initial 2 years and subsequently every 6 months in the third year. Patients were monitored for a period of 3 years, or until their death. Physical examinations and hematology tests were performed every 3–6 months for 1–2 years, and every 6–12 months for 3 years. Computed tomography (CT) scans of the chest and abdomen were conducted every 6–12 months during the first 2 years, followed by annual scans up to the third year.

### 2.3 Statistical analysis

The determination of the sample size was derived from the calculation related to the 3‐year overall survival (OS). Based on the results of the adjuvant chemotherapy with S‐1 plus cisplatin^17^ and the CLASS trial,^8^ in which patients received curative D2 gastrectomy through open or laparoscopic surgery, the 3‐year OS of the S‐1 plus cisplatin (84.5%) and CAPOX (83%) regimens was adopted. The test performance was set to 80%. The two‐sided significance level was set as 0.05. The noninferiority margin was set as 0.15. The data were processed in accordance with modified intent‐to‐treat analysis (mITT) analysis. OS was defined as the time from the date of random assignment to the date of death from any cause. DFS was defined as the duration from randomization to the occurrence of the first recurrence, development of second primary tumors, or death from any cause. For quantitative variables, data were converted into categorical data. Categorical data were presented as numbers (percentages) and subjected to analysis through chi‐squared and Fisher's exact tests. The prognostic impact of the two regimens on OS was assessed using univariate Cox regression analysis. Survival curves were assessed utilizing the Kaplan–Meier method and compared through the log‐rank (Mantel–Cox) test. Statistical tests were at a two‐sided 0.05 significance level. All statistical analyses were performed using SPSS software (version 26.0).

## 3 RESULTS

### 3.1 Patient characteristics

**FIGURE 1:**
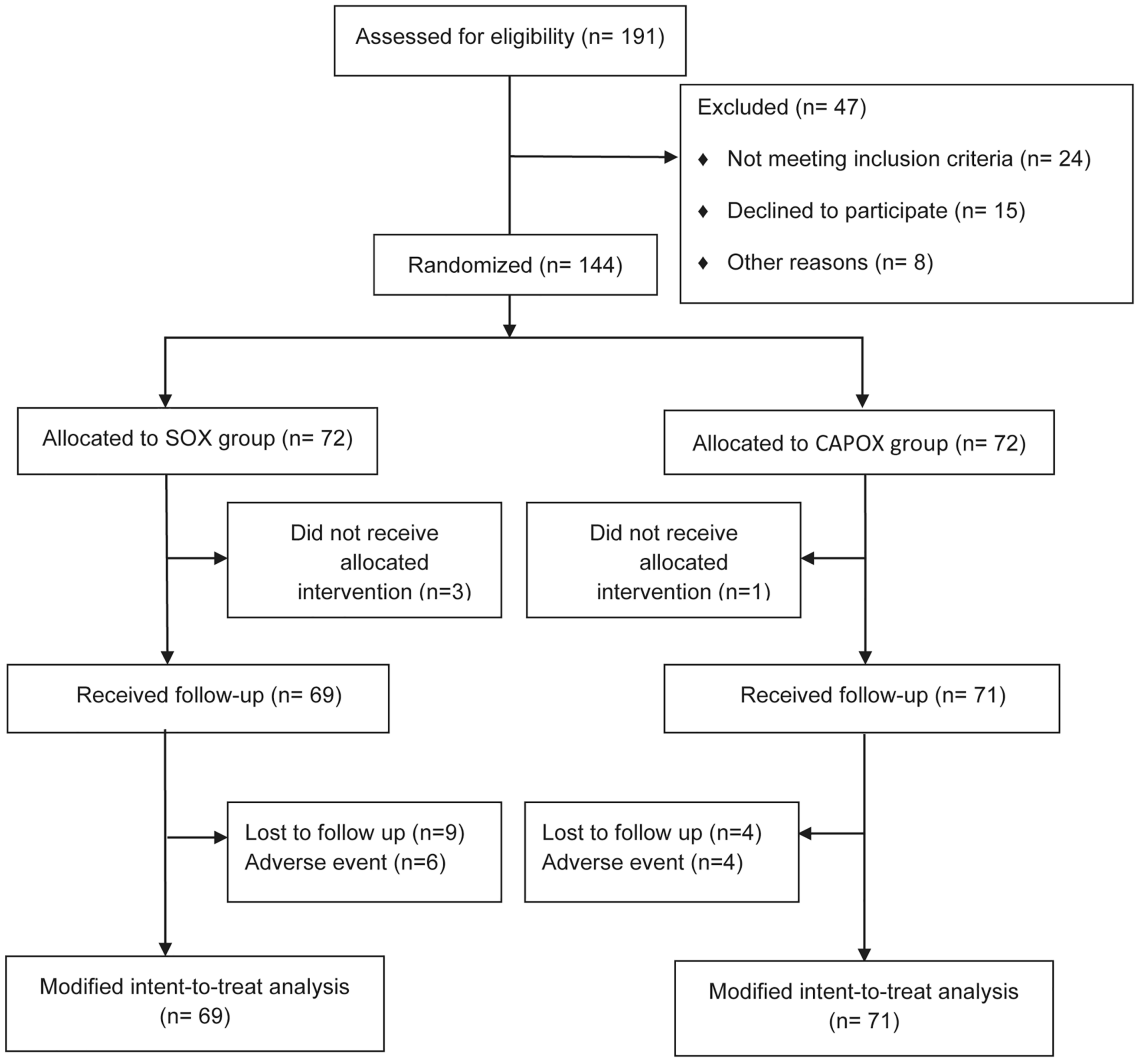
The trial profile for the adjuvant chemotherapy in gastric cancer. CAPOX, capecitabine plus oxaliplatin; SOX, S‐1 plus oxaliplatin.

Between May 2018 and June 2019, 144 patients were enrolled from three centers in China. A total of 140 patients (69 in the SOX group and 71 in the CAPOX group) satisfied the eligibility criteria and were included in the mITT analysis sets (Figure 1). The median age of the patients was 61 years (range: 23–76 years). There were no significant differences in general parameters between SOX and CAPOX groups (Table 1). The clinicopathological information is summarized in Table 2. Distal and total gastrectomy were performed in 73 (52.1%) and 67 patients (47.9%), respectively. Reconstruction was mainly performed by Billroth II reconstruction (32.1%) and Roux‐en‐Y esophagojejunostomy (35.0%). The pathology grade was mainly poorly differentiated (44.3%) or moderately differentiated (54.3%).

**TABLE 1:** Baseline patient characteristics.

	All (*n* = 140)		SOX group (*n* = 69)		CAPOX group (*n* = 71)		*p‐*Value
Sex							0.716
Male	97	69.3%	49	71.0%	48	67.6%	
Female	43	30.7%	20	29.0%	23	32.4%	
Age							0.310
≤60	66	47.1%	36	52.2%	30	42.3%	
>60	74	52.9%	33	47.8%	41	57.7%	
BMI^a^							0.404
≤18.4	7	5.0%	3	4.3%	4	5.6%	
18.5–23.9	77	55.0%	34	49.3%	43	60.6%	
24.0–27.9	34	24.3%	18	26.1%	16	22.5%	
≥28.0	22	15.7%	14	20.3%	8	11.3%	
ECOG							1.000
0	66	47.1%	33	47.8%	33	46.5%	
1	74	52.9%	36	52.2%	38	53.5%	
Hypertension							0.725
Yes	50	35.7%	26	37.7%	24	33.8%	
No	90	64.3%	43	62.3%	47	66.2%	
Diabetes							0.695
Yes	34	24.3%	18	26.1%	16	22.5%	
No	106	75.7%	51	73.9%	55	77.5%	
Smoking history							0.863
Yes	86	61.4%	43	62.3%	43	60.6%	
No	54	38.6%	26	37.7%	28	39.4%	
Alcohol abuse							0.498
Yes	76	54.3%	35	50.7%	41	57.7%	
No	64	45.7%	34	49.3%	30	42.3%	

Abbreviations: BMI, body mass index; CAPOX, capecitabine plus oxaliplatin; ECOG, Eastern Cooperative Oncology Group; SOX, S‐1 plus oxaliplatin.

^a^ Fisher's exact tests were performed.

**TABLE 2:** Surgical and pathological results of patients.

	SOX group (*n* = 69)		CAPOX (*n* = 71)		*p‐*Value
Type of gastrectomy					0.866
Partial	35	50.7%	38	53.5%	
Total	34	49.3%	33	46.5%	
Reconstruction					0.765
BI	4	5.8%	2	2.8%	
BII	21	30.4%	24	33.8%	
R‐Y EJ	26	37.7%	23	32.4%	
R‐Y GJ	8	11.6%	12	16.9%	
Overlap	10	14.5%	10	14.1%	
Tumor position					0.683
Gastric antrum	34	49.3%	33	46.5%	
Body	24	34.8%	28	39.4%	
Fundus	3	4.3%	5	7.0%	
Body and fundus	8	11.6%	5	7.0%	
Tumor size (cm)					0.126
≤5	55	79.7%	48	67.6%	
>5	14	20.3%	23	32.4%	
T stage					0.663
T2	9	13.0%	10	14.1%	
T3	11	16.0%	12	16.9%	
T4a	41	59.4%	36	50.7%	
T4b	8	11.6%	13	18.3%	
N stage					0.515
N0	19	27.5%	18	25.4%	
N1	8	11.6%	13	18.3%	
N2	19	27.5%	12	16.9%	
N3a	13	18.8%	16	22.5%	
N3b	10	14.5%	12	16.9%	
TNM stage^a^					0.801
I	8	11.6%	6	8.5%	
II	20	29.0%	20	28.2%	
III	41	59.4%	45	63.4%	
Differentiation^a^					0.982
Poor	30	43.5%	32	45.1%	
Moderate	38	55.1%	38	53.5%	
Well	1	1.4%	1	1.4%	
CK					0.695
Negative	18	26.1%	16	22.5%	
Positive	51	73.9%	55	77.5%	
CEA					0.086
Negative	47	68.1%	38	53.5%	
Positive	22	31.9%	33	46.5%	
Ki67					0.076
+	14	20.3%	9	12.7%	
++	31	44.9%	24	33.8%	
+++	24	34.8%	38	53.5%	

*Note*: “+” represented a positivity rate of less than 30%; “++” meant 30%–60%; “+++” meant >60%.

Abbreviations: BI, Billroth I; BII, Billroth II; CAPOX, capecitabine plus oxaliplatin; CEA, carcinoembryonic antigen; CK, cytokeratin; R‐Y EJ, Roux‐en‐Y and esophagojejunostomy; R‐Y GJ, Roux‐en‐Y and gastrojejunostomy; SOX, S‐1 plus oxaliplatin.

^a^ Fisher's exact tests were performed.

### 3.2 Efficacy

**FIGURE 2:**
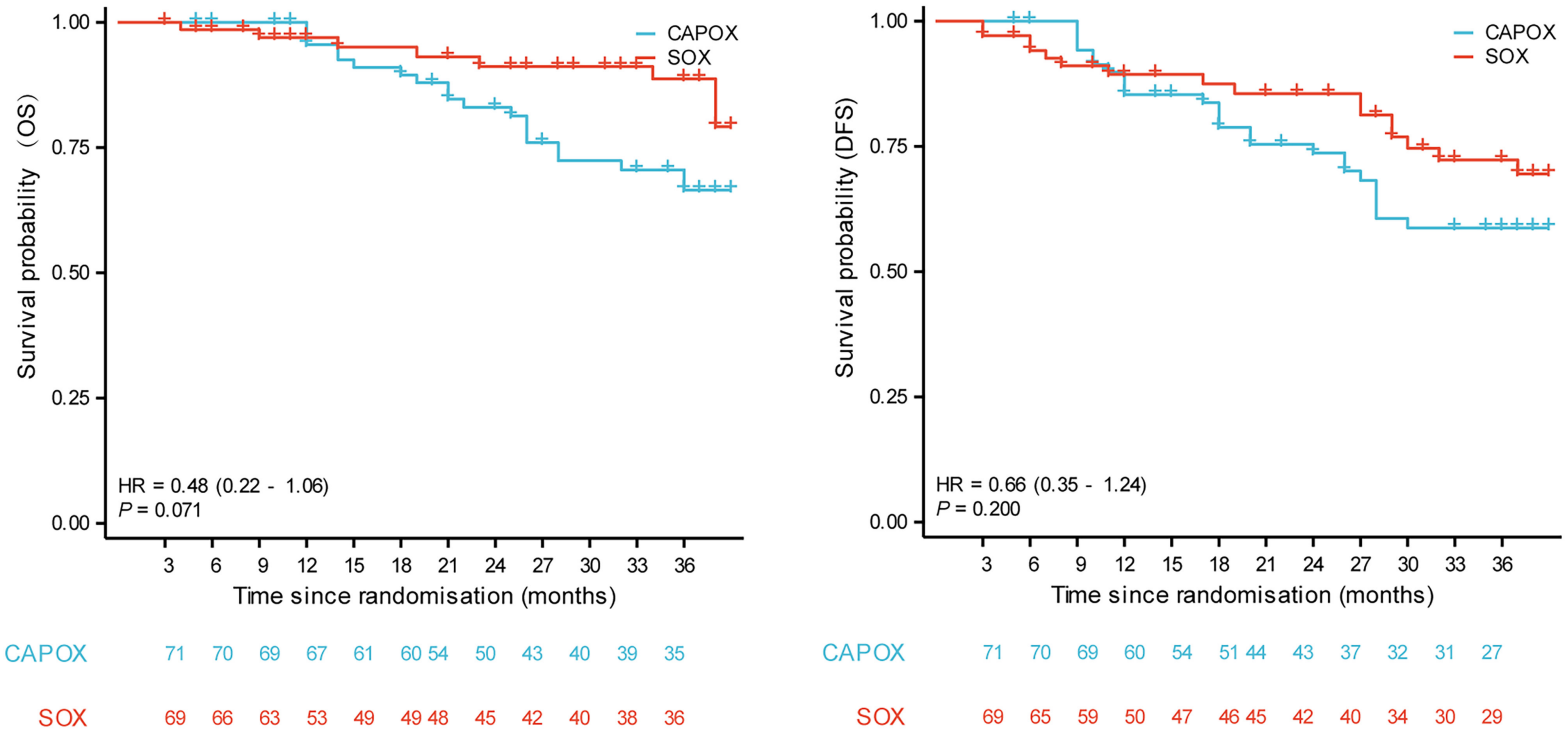
OS and DFS for patients treated with SOX and CAPOX. CAPOX, capecitabine plus oxaliplatin; DFS, disease‐free survival; OS, overall survival; SOX, S‐1 plus oxaliplatin.

**FIGURE 3:**
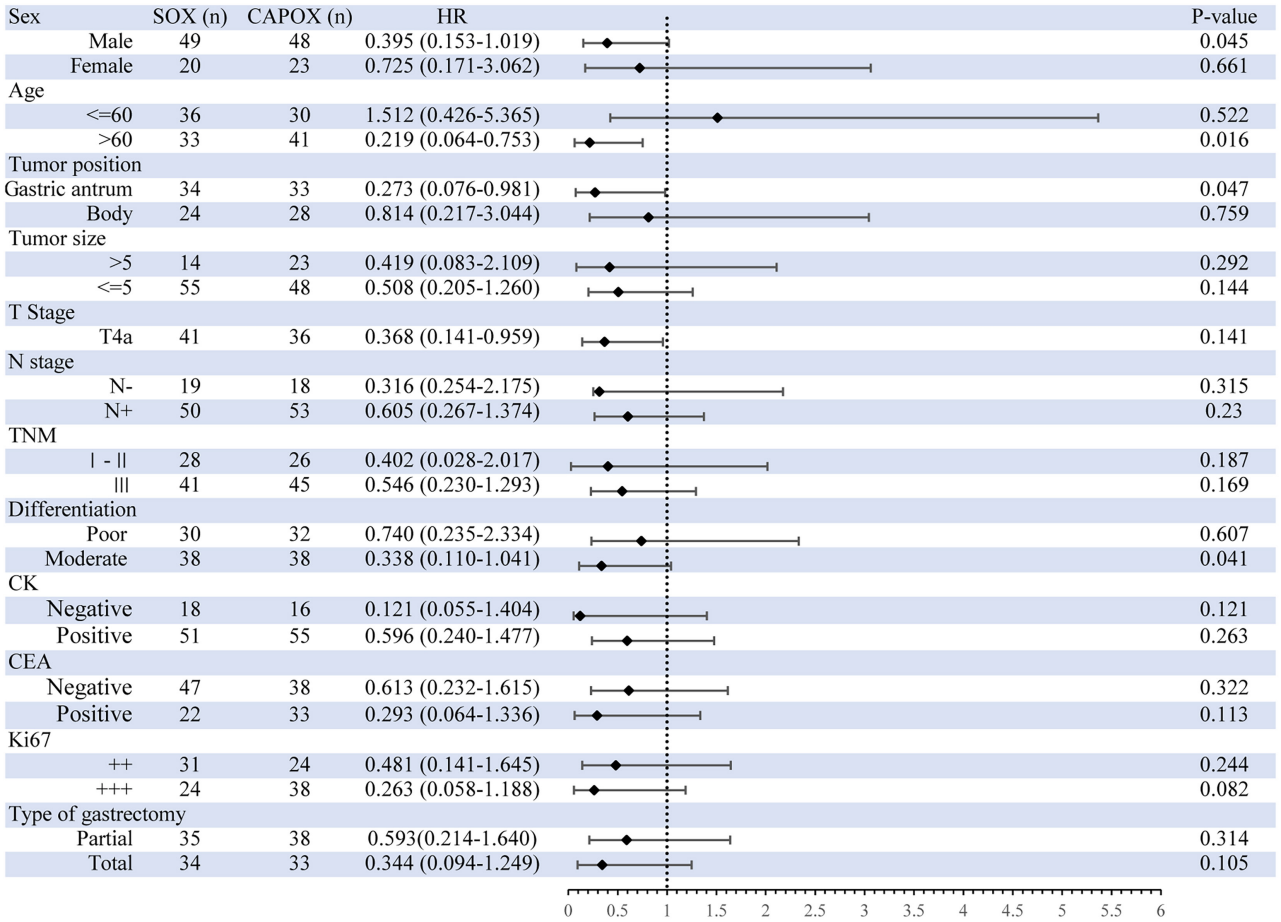
Subgroup analyses of 3‐year OS by stratification in the intention‐to‐treat population. CAPOX, capecitabine plus oxaliplatin; CEA, carcinoembryonic antigen; CK, cytokeratin; HR, hazard ratio; SOX, S‐1 plus oxaliplatin. CK, CEA, and Ki‐67 were assessed by immunohistochemistry (IHC) staining. “−” meant negative; “+” represented a positivity rate of less than 30%; “++” meant 30%–60%; “+++” meant >60%.

The enrolled 140 patients were followed up for a minimum period of 36 months. By June 2022, the estimated 3‐year OS in the final enrollment population were 43.7 months (95% CI 41.1–46.4) and 38.2 months (95% CI 35.3–41.2) in the SOX and CAPOX groups, respectively. The HR for OS events in the SOX group compared to the CAPOX group was 0.48 (95% CI 0.22–1.06; *p* = 0.071, Figure 2). The 3‐year DFS were 38.2 months (95% CI 34.8–41.6) and 34.9 months (95% CI 31.4–38.4) in the SOX and CAPOX groups, respectively. The HR for DFS events in the SOX group compared to the CAPOX group was 0.66 (95% CI 0.35–1.24; *p* = 0.200). Subgroup analysis of OS is shown in Figure 3. Patients in both the SOX and CAPOX groups were stratified based on sex, age, tumor position, tumor size, type of gastrectomy, TNM staging, differentiation degree, and pathologic results. Age and tumor size were dichotomized according to their median. Some subgroups were not included in the analysis due to the small number of patients, such as the T‐stage analysis. Some significant differences were observed in OS in patients classified according to sex, age, tumor position, and degree of differentiation. In the subgroup analysis, for male patients (*p* = 0.045), age older than 60 years (*p* = 0.016), tumors in the gastric antrum (*p* = 0.047), and moderately differentiated tumors (*p* = 0.041), the SOX group exhibited a superior overall survival (OS) rate compared to the CAPOX group.

### 3.3 Adverse events

In total, 130 (92.9%) patients in the mITT analysis set completed the planned chemotherapy with a full dose of the respective chemotherapy regimen. Six patients in SOX group and 4 patients in the CAPOX group changed treatment plans due to adverse events. Adverse events of all grades were documented in 34 (49.2%) out of 69 patients in the SOX group and 41 (57.7%) out of 71 patients in the CAPOX group (Table 3). Eleven (15.9%) patients in the SOX group and 13 (18.3%) patients in the CAPOX group had Grade 3 or 4 adverse events. There were no reported deaths related to the treatment. The most common adverse events were gastrointestinal and hematologic symptoms. The gastrointestinal symptoms in SOX and CAPOX groups predominantly included anorexia (18.8% vs. 19.7%), nausea (13.0% vs. 21.1%), vomiting (17.4% vs. 19.7%), and diarrhea (11.6% vs. 12.7%). The adverse events were not significantly different between the two groups. Neutropenia was the most common Grade 3 or 4 adverse events (8 [11.6%] out of 69 patients in the SOX group, and 9 [12.7%] out of 71 patients in the CAPOX group). There were no significant differences between the two groups in terms of Grade 3 and 4 adverse events.

**TABLE 3:** Summary of adverse events.

	SOX group (*n* = 69)		CAPOX group (*n* = 71)		*p‐*value	
	All grades	Grade 3–4	All grades	Grade 3–4	All grades	Grade 3–4
Anorexia	13 (18.8%)	2 (2.9%)	14 (19.7%)	1 (1.4%)	0.533	0.617
Nausea	9 (13.0%)	3 (4.3%)	15 (21.1%)	2 (2.8%)	0.263	0.678
Vomiting	12 (17.4%)	5 (7.2%)	14 (19.7%)	1 (1.4%)	0.829	0.113
Diarrhea	8 (11.6%)	1 (1.4%)	9 (12.7%)	0 (0.0%)	0.525	0.493
Sensory neuropathy	8 (11.6%)	1 (1.4%)	14 (19.7%)	1 (1.4%)	0.246	0.745
Fatigue	13 (18.8%)	1 (1.4%)	15 (21.1%)	5 (7.0%)	0.834	0.209
Leukopenia	13 (18.8%)	2 (2.9%)	13 (18.3%)	2 (2.8%)	1.000	1.000
Neutropenia	18 (26.1%)	8 (11.6%)	22 (31.0%)	9 (12.7%)	0.577	0.525
Anemia	9 (13.0%)	1 (1.4%)	9 (12.7%)	2 (2.8%)	0.574	0.511
Thrombocytopenia	11 (15.9%)	2 (2.9%)	13 (18.3%)	2 (2.8%)	0.442	0.679
Total bilirubin increased	15 (21.7%)	0 (0.0%)	12 (16.9%)	3 (4.2%)	0.524	0.245
AST/ALT increased	6 (8.7%)	1 (1.4%)	8 (11.3%)	0 (0.0%)	0.780	0.493
Creatinine increased	8 (11.6%)	0 (0.0%)	6 (8.5%)	1 (1.4%)	0.584	1.000

Abbreviations: CAPOX, capecitabine plus oxaliplatin; SOX, S‐1 plus oxaliplatin.

### 3.4 Patterns of recurrence

In all patients who received chemotherapy, the anastomosis (7.8%), peritoneum (6.4%), and lymph nodes (6.4%) were the most common sites of recurrence (Table 4). Earlier disease progression and death were observed in the patients with recurrence or metastasis. Among the 13 patients who experienced recurrence after the SOX regimen, anastomosis (5, 7.2%) was the most common recurrence site, followed by lymph nodes (4, 5.8%), peritoneum (4, 5.8%), liver (3, 4.3%), and pancreas (1, 1.4%). Consistent with the SOX regimen, among the 17 patients who experienced recurrence in the CAPOX group, anastomotic recurrence (6, 8.5%) and peritoneum metastasis (5, 7.0%) also had the highest incidences. No statistically significant difference was observed in the distribution of recurrence sites between the two chemotherapy regimens.

**TABLE 4:** Site of recurrence.

Recurrence site	Total patients (*n* = 140)	SOX (*n* = 69)		CAPOX (*n* = 71)	
Anastomosis	11	5	7.2%	6	8.5%
Peritoneum	9	4	5.8%	5	7.0%
Lymph node	9	4	5.8%	5	7.0%
Liver	5	3	4.3%	2	2.8%
Pancreas	1	1	1.4%	0	0.0%
Other	3	1	1.4%	2	2.8%

Abbreviations: CAPOX, capecitabine plus oxaliplatin; SOX, S‐1 plus oxaliplatin.

## 4 DISCUSSION

In this multicenter cohort study, patients in both the SOX and CAPOX groups exhibited similar 3‐year overall survival (OS) and disease‐free survival (DFS), aligning with outcomes reported in other clinical trials.^18^, ^19^ No significant difference in survival was observed between the two treatment regimens. In the subgroup analysis, the group of patients with male sex, age >60 years, tumors in the gastric antrum, and moderately differentiated tumors showed significantly better OS for the SOX regimen. To our knowledge, this clinical trial represents the inaugural assessment of survival outcomes for SOX and CAPOX in patients with gastric cancer who underwent laparoscopic D2 gastrectomy.

In the ARTIST 2 trial, patients with D2 gastrectomy, Stage II/III, and adjuvant SOX benefited from better 3‐year DFS compared with S‐1 monotherapy (74.3% vs. 63.8%). In our study, patients who received the SOX regimen demonstrated a 3‐year DFS rate of 76.8%, which showed a certain degree of improvement compared to the ARTIST 2 trial. A possible reason for this was the lower proportion of patients with Stage III in our study (59.4% vs. 72%). In the CLASSIC trial,^8^, ^20^ both the 3‐year and 5‐year DFS rates showed improvement in the CAPOX group compared to the surgery‐only group. In the present study, a lower 3‐year DFS was observed compared with the results of the CLASSIC trial. This discrepancy could be attributed to several reasons. The median patient age was 56.1 in the CLASSIC trial which was lower than that in our study. Fewer patients had pathologic stage T4 or N3 in the CAPOX group in the CLASSIC trial than in our study (49 [69.0%] out of 71 patients with pT4 and 28 [39.4%] patients with pN3). Thus, the difference in the distribution of enrolled patients may be the reason for the difference between the results of our study and those of the previous studies. A comparison between SOX and CAPOX regimens has also been explored in some studies. In a survival analysis of the results of the SOXaGC and J‐CLASSIC trials, similar efficacy was found between the two regimens for Stage III patients who underwent D2 gastrectomy.^19^ In the RESOLVE trial, the adjuvant‐SOX regimen was confirmed to be non‐inferior to adjuvant CAPOX in patients with cT4a/4b.^9^

However, previous studies on SOX or CAPOX focused on chemotherapy regimens alone without considering the surgical procedure. For advanced GC, laparoscopic surgery was gradually being considered a reliable treatment choice.^10^, ^11^ Whether the use of energy platforms such as ultrasonic scalpels and electrical equipment, and the establishment of pneumoperitoneum during laparoscopic surgery increases the risk of tumor cell dissemination and implantation in the abdominal cavity remains controversial.^12^ Gimeracil in S‐1 could improve the antitumor efficacy of tegafur by effectively slowing down the decomposition of fluorouracil, prolonging the drug exposure time, and increasing the concentration of drugs locally through the inhibition of dihydropyrimidine dehydrogenase.^21^ Thus, there was a difference in the postoperative benefits of the two treatment options in patients underwent laparoscopic surgery. In this study, our findings were in accordance with those of the previous studies; the 3‐year OS and DFS were not significantly different in the two regimens.

Although there was no statistical difference in survival outcomes between the SOX and CAPOX groups, the SOX group appeared to be non‐inferior to the CAPOX group. We speculated that it may be that the SOX regimen can bring survival benefits to some characteristic populations. Survival outcomes in the subgroup analysis showed that adjuvant SOX seemed to be more favorable for patients with male sex, age >60 years, tumors in the gastric antrum, and moderately differentiated tumors. In a study on the elderly population, S‐1 monotherapy had a significantly better DFS rate than the CAPOX regimen.^22^ Genetic characteristics and drug response were possible reasons. The findings of one study indicated that integrating sex‐related factors into cancer patient care improves outcomes.^23^ They proposed that chemotherapy exhibits significantly higher toxicity in women compared to men, attributed to the slower clearance of drugs in women. Contrary to our results, another study showed that female patients seemed to gain more survival benefits from the SOX regimen.^19^ Tumor cell differentiation increased the sensitivity of tumor cells to chemotherapy.^24^ In our study, patients with moderately differentiated tumors gained survival benefits from SOX regimen. This may be due to the high degree of malignancy of poorly differentiated tumors, which negated the superiority of the chemotherapy. The influence of tumor position on chemotherapy responses to SOX and CAPOX regimens has not been reported. However, it was imperative to acknowledge the limitations for the subgroup analysis in our study. It became apparent that certain subgroups had limited sample sizes, leading them inadequate for conducting robust analysis. Despite our efforts to explore the difference between the two groups, the small sample sizes in some subgroups hampered our ability to draw conclusive findings. In the future, we will need more samples for multivariable Cox regression analysis and other considerations to guide the selection of chemotherapy regimens for patients.

The toxicity of chemotherapeutic agents commonly impacts the course of treatment. In a Phase II study of the SOX regimen for patients with Stage III GC, all patients experienced adverse events and 62.9% of patients experienced Grade 3 or 4 adverse events.^16^ In the safety analysis of this study, no significant difference in adverse events was identified between the two treatment arms. The completion rates for the SOX group and the CAPOX group were 87.5% and 93.1%, respectively. The reasons for reducing or discontinuing the SOX regimen were severe vomiting and neutropenia. For CAPOX regimen was bone marrow suppression. The highest incidence of Grade 3 or 4 adverse events was observed for neutropenia, reaching 12.1%. This was also the most common cause of changes in the prespecified treatment protocol. In general, laparoscopic surgery did not markedly affect the adverse events associated with SOX and CAPOX regimens in patients with GC.

In the patterns of recurrence, the anastomosis was the most common site of recurrence. The two groups showed no significant statistical difference in the recurrence sites. Compared to early GC, the anastomotic recurrence rate may be higher in advanced GC. Moreover, in this study, some cases of extra gastric recurrence with infiltration of the remnant stomach were classified as anastomotic recurrence. That maybe caused the high proportion of anastomotic recurrences in our study.

## 5 CONCLUSIONS

Postoperative chemotherapy with SOX and CAPOX has the same efficacy in patients with GC who undergo laparoscopic D2 gastrectomy. In addition, differences in treatment efficacy with respect to age, sex, tumor position, and tumor differentiation degree may be useful to consider when determining the postoperative treatment regimens in these patients.

## AUTHOR CONTRIBUTIONS

**Xin Liu:** Conceptualization (equal); data curation (equal); software (equal); visualization (equal); writing – original draft (equal). **Yongjia Yan:** Formal analysis (equal); funding acquisition (equal). **Li Lu:** Investigation (equal); methodology (equal); project administration (equal). **Yang Liu:** Resources (equal); software (equal). **Jun Ma:** Methodology (equal); project administration (equal). **Xi Wang:** Formal analysis (equal); validation (equal); visualization (equal). **Daohan Wang:** Formal analysis (equal); software (equal); supervision (equal). **Bang Liu:** Methodology (equal); writing – original draft (equal). **Zhuo Liu:** Validation (equal); visualization (equal). **Xueying Zhou:** Methodology (equal); project administration (equal); writing – original draft (equal). **He Cui:** Data curation (equal); formal analysis (equal); resources (equal). **Zhicheng Zhao:** Formal analysis (equal); supervision (equal); validation (equal). **Chuan Li:** Data curation (equal); resources (equal); software (equal). **Jian Liu:** Supervision (equal); validation (equal); visualization (equal). **Weidong Li:** Methodology (equal); software (equal); writing – review and editing (equal). **Qing‐Xing Huang:** Methodology (equal); project administration (equal). **Qun Zhao:** Supervision (equal); validation (equal). **Tong Liu:** Supervision (equal); validation (equal). **Weihua Fu:** Conceptualization (equal); data curation (equal); project administration (equal); writing – review and editing (equal).

## FUNDING INFORMATION

This work was supported by grants from the Tianjin Key Medical Discipline (Specialty) Construction Project (TJYXZDZK‐005A), Tianjin Medical University Clinical Research Fund, Fundamental Research Cooperation Program of Beijing‐Tianjin‐Hebei Region of Natural Science Foundation of Tianjin (22JCZXJC00140); Tianjin Major Science and Technology Project (21ZXJBSY00110).

## CONFLICT OF INTEREST STATEMENT

The authors declare that they have no conflict of interest to declare.

## ETHICS STATEMENT

Approval of the research protocol by an Institutional Review Board: This study was approved by the Institutional Ethical Boards of Tianjin Medical University General Hospital (IRB2018‐035‐01).

Informed consent: All patients agreed to participate in the study and signed informed consent forms.

Registry and the registration no. of the study/trial: The study is registered at Chinese Clinical Trial Registry (Chi‐CTR‐IPR‐17013680).

## Supporting information


Doc. S1.

## Data Availability

Raw data are available from corresponding author with plausible reasons.
