# Supplementary material for: Comparison of SOX and CAPOX in patients with advanced gastric cancer after laparoscopic D2 gastrectomy: A randomized controlled trial
Source: Cancer Med. 2024 Jun 3;13(11):e7326. doi: 10.1002/cam4.7326 (PMC11145022; doi:10.1002/cam4.7326)
Supplement: Supplementary file 1 — Doc. S1. [file CAM4-13-e7326-s001.docx]

**Surgical procedures of laparoscopic D2 gastrectomy.**

The assessment of the resection status is determined through intraoperative assessment by the surgeon and postoperative pathological results. All patients underwent laparoscopic D2 gastrectomy under general anesthesia with adequate preoperative preparation. Depending on the location of the tumor, different surgical methods were selected: for distal GC, laparoscopic distal gastrectomy was selected; for proximal GC and gastric body cancer, laparoscopic total gastrectomy was selected. If R0 resection could not be achieved through D2 dissection during abdominal exploration, the patient was withdrawn from the trial. The operation was performed by a team of medical doctors and the operation standards were uniform and controllable. The number of abdominal wall holes during the operation was 5-6, which could not be increased or decreased. The length of the auxiliary abdominal incision was ≤6cm. The surgical procedure was as follows: 1) establish pneumoperitoneum, explore the abdominal cavity, and determine the surgical method; 2) resect the greater omentum, remove the greater curvature lymph nodes (group 4d, 4sb, total gastrectomy dissection 4sa group), cut off the right gastroepiploic vessel at the root, and remove the subpyloric lymph nodes (group 6); 3) lift the stomach, cut off the right gastric blood vessel at the root, and clean the upper pyloric lymph nodes (group 5); 4) separate along the upper border of the pancreas, cut off the left gastric vessel at the root, and clean the lymph nodes around the left gastric artery (group 7); clean the lymph nodes of the splenic artery (group 11p, 11d) and the lymph nodes in front of the common hepatic artery (group 8a) to the left and right respectively and lymph nodes around the celiac artery (group 9); 5) dissect lesser curvature lymph nodes (group 3) and right cardia lymph nodes (group 1) at the back of the stomach; 6) pull the stomach down, dissect the hepatoduodenal ligament lymph nodes (group 12a), cut the hepatogastric ligament along the liver, and reach the right side of the cardia; 7) determine the extent of resection according to the tumor location, resection of the specimen, and reconstruction of the digestive tract.
